# Interactions of the Algicidal Bacterium *Kordia algicida* with Diatoms: Regulated Protease Excretion for Specific Algal Lysis

**DOI:** 10.1371/journal.pone.0021032

**Published:** 2011-06-17

**Authors:** Carsten Paul, Georg Pohnert

**Affiliations:** Institute for Inorganic and Analyticial Chemistry, Department for Bioorganic Analytics, Friedrich Schiller University Jena, Jena, Germany; US Dept. of Agriculture – Agricultural Research Service (USDA-ARS), United States of America

## Abstract

Interactions of planktonic bacteria with primary producers such as diatoms have great impact on plankton population dynamics. Several studies described the detrimental effect of certain bacteria on diatoms but the biochemical nature and the regulation mechanism involved in the production of the active compounds remained often elusive. Here, we investigated the interactions of the algicidal bacterium *Kordia algicida* with the marine diatoms *Skeletonema costatum*, *Thalassiosira weissflogii*, *Phaeodactylum tricornutum*, and *Chaetoceros didymus*. Algicidal activity was only observed towards the first three of the tested diatom species while *C. didymus* proved to be not susceptible. The cell free filtrate and the >30 kDa fraction of stationary *K. algicida* cultures is fully active, suggesting a secreted algicidal principle. The active supernatant from bacterial cultures exhibited high protease activity and inhibition experiments proved that these enzymes are involved in the observed algicidal action of the bacteria. Protease mediated interactions are not controlled by the presence of the alga but dependent on the cell density of the *K. algicida* culture. We show that protease release is triggered by cell free bacterial filtrates suggesting a quorum sensing dependent excretion mechanism of the algicidal protein. The *K. algicida* / algae interactions in the plankton are thus host specific and under the control of previously unidentified factors.

## Introduction

Diatoms (Bacillariophyceae) are very abundant unicellular microalgae in marine and freshwater ecosystems and are highly ecologically relevant because of their position at the bottom of the marine food web [1]. Different diatom species can occur in dense blooms and dominate the phytoplankton community during short or prolonged periods. Because of their ecological importance, understanding the factors that limit diatom growth and proliferation is crucial. These can include abiotic factors such as extreme light or temperature conditions or nutrient limitation [2]. But also biotic factors such as grazing by zooplankton [3], [4], allelopathic effects of other phytoplankton species [5], or viral infections can have a negative impact on diatoms [6], [7]. It is also documented that bacteria can even control bloom termination processes [8], [9].

In terms of cell numbers marine bacteria are even more abundant than diatoms and by utilization of organic matter they also play a key role in plankton communities [10]. Interactions between phytoplankton and bacteria have gained increasing attention as the relevance of the microbial loop for plankton communities becomes more evident [11], [12], [13]. Bacteria can act synergistically with diatoms and symbiotic interactions have been reported from several systems [11], [14], [15]. But bacteria can also control algal populations e.g. by inhibiting growth of diatoms and other phytoplankton members or by active lysis of algal cells [16], [17], [18]. Bacterial inhibition of algal growth either requires direct cell contact [19] or can be mediated by excreted extracellular substances [18], [20]. Inhibitory interactions between bacteria and phytoplankton are mostly investigated with the goal of finding a biological control for harmful algal blooms [21], [22]. In contrast, only few ecological studies on the bloom termination of non-harmful plankton species exist [12], [18]. Besides few exceptions the identity of the compounds or enzymes responsible for the algicidal effect remains unknown. Lee *et al.* [20] demonstrated that *Pseudoalteromonas* sp. produces a high molecular weight extracellular protease which is able to inhibit the growth of the diatom *Skeletonema costatum*. But lower molecular weight algicidal compounds, such as rhamnolipid biosurfactants from *Pseudomonas aeruginosa* or the pigment prodigiosin from the bacterium *Hahella chejuensis* have also been identified [23], [24].

The regulation of the production of such inhibitory compounds is mostly unknown. An exception is the report on genes potentially involved in prodigiosin biosynthesis [25]. Generally, bacterial production of inhibitory substances can be regulated by external factors which might also be a relevant mechanism for planktonic species. Examples from the terrestrial environment include mechanisms where secretion of active metabolites occurs only in the presence of the host or where the release of active compounds is dependent on the cell density of the bacteria [26]. The latter process is known as quorum sensing (QS). QS is a process governed by small molecules such as acyl homoserine lactones or peptides that are excreted from bacteria. Reception of such metabolites allows bacteria to determine the local density of their population and to regulate gene expression. These changes in gene expression can result in a variety of physiological changes like the onset of bioluminescence, antibiotic synthesis or extracellular enzyme production [26].

In a screening of algicidal bacteria the aerobic, Gram-negative, non-motile *Kordia algicida* was isolated during a bloom of the cosmopolitan diatom *Skeletonema costatum*. The bacterium was able to kill *S. costatum* and also exhibited algicidal activity against other microalgae in co-culture experiments [27]. The genome sequencing of *K. algicida* is underway and interestingly, several genes coding for proteases have been identified and deposited in the databases. We decided to investigate *K. algicida*/diatom interactions in more detail. We reasoned that for any bacterium in the dilute matrix of the plankton, secretion of secondary metabolites or proteins that mediate lysis of diatoms is costly and thus we proposed the hypothesis that algicidal activity is controlled by biotic signals in the *K. algicida*/*S. costatum* system.

In this study we show that the algicidal bacterium *K. algicida* relies on diffusible enzymes >30 kDa to interfere with algal growth. We show that the activity is specific for certain diatoms, while others are not susceptible. Furthermore we show that the excretion of active proteases is not regulated by the presence of a co-cultured diatom species but is rather dependent on the bacteria cell density in a process that bears the hallmarks of quorum sensing.

## Methods

### Algal and bacteria culturing

The Gram-negative marine bacterium *Kordia algicida* strain OT-1 was originally isolated from a *Skeletonema costatum* bloom [27] and was obtained from the NITE Biological Resource Center (NBRC 100336). Cultures were grown at 15°C under constant shaking (90–100 rpm min^−1^) in autoclaved ZoBell medium (5 g bacto peptone, 1 g yeast extract, 10 mg FePO_4_, 34 g of Instant Ocean in 1 L bidistilled water) [28]. Dense cultures were used to prepare glycerol stock cultures (20 vol. %). Before each set of experiments a new culture was started from the glycerol stock.

Non-axenic *S. costatum* (RCC75) and *Thalassiosira weissflogii* (RCC76) were obtained from the Roscoff Culture Collection, France. *Phaeodactylum tricornutum* (UTEX 646) was obtained from the Culture Collection of Algae in Austin, TX, USA. *Chaetoceros didymus* (CH5) was isolated by S. Poulet, Station Biologique, Roscoff, France and is maintained in our culture collection. The strains were cultivated under a 14/10 hours light/dark cycle with 40–45 µmoles photons s^−1^ m^−2^ at 15°C in artificial seawater prepared according to Maier and Calenberg at a pH of 7.8 [29]. The nutrient concentrations were 620 µM nitrate, 14.5 µM phosphate and 320 µM silicate.

### Estimating bacterial and algal growth

The optical density (OD) of *K. algicida* cultures was measured with a Specord M42 UV-vis spectrophotometer by Carl Zeiss (Jena Germany) at a wavelength of 550 nm. Bacterial growth rate was estimated graphically by plotting measured OD values on a logarithmic scale. Time points that showed a linear increase were used to perform an exponential regression with OD_2_ =  OD_1_ e^µt^ wereµ represents the bacterial growth rate and OD_1_ and OD_2_ represent the optical densities at time point 1 and 2, respectively.

Algal growth was determined by measuring the *in vivo* chlorophyll a fluorescence using 300 µL of each culture in 96 well plates or 1.5 mL in 24 well plates. The fluorescence was measured with a Mithras LB 940 plate reader by Berthold Technologies (Bad Wildbad, Germany). Cell density was determined using a Fuchs-Rosenthal hematocytometer with an upright microscope (Leica DM 200, Leica, Germany).

### Generation of cell free bacterial and co-culture filtrates

Exponentially growing *K. algicida* was inoculated into a 10∶1 (vol. %) mixture of artificial seawater and ZoBell medium. After the culture reached an optical density (OD) >0.32 one mL was diluted into 50 mL of seawater. After 24 hours the cultures were gently filtered through a 0.22 µm sterile polyethersulfone (PES) filter (Carl Roth; Karlsruhe, Germany). To obtain a cell free filtrate of *S. costatum*/*K. algicida* co-cultures, 1 mL of bacterial culture in 10∶1 seawater : ZoBell media (OD> 0.32) was inoculated with 50 mL of an exponentially growing *S. costatum* culture (ca. 1.5 10^6^ cells mL^−1^) and grown for 24 h before filtration as described above.

### Monitoring algicidal activity

We inoculated 1.125 mL of the filtrate (see above) with 375 µL of the respective exponentially growing diatom culture in the wells of 24 well plates. For controls aliquots of 375 µL of the same starting cultures were diluted with 1.125 mL of artificial seawater. Plates were cultured under the previously mentioned conditions and measurements were performed over regular time intervals. The *in vivo* fluorescence of chlorophyll a of all cultures was measured as indicator for algal growth.

### Size fractionation

Size fractionation experiments were performed with a filtrate of a co-culture of *S. costatum* and *K. algicida* as well as with filtrates of mono-cultures of these species (see above). A volume of 15 mL of the respective filtrates was fractionated using Amicon Ultra centrifugal filter units with a molecular weight cut off of 30 kDa (Millipore, Billerica, MA, USA) as described in the manufacturer’s instructions. The high molecular weight fraction was diluted to 1.5 mL with artificial seawater. The biological activity of the filtrates was monitored in 96 well plates by inoculating 240 µL of raw or fractionated filtrates with 60 µL of exponentially growing *S. costatum.*

### Heat inactivation of filtrates

Active cell free filtrates of *S. costatum*, *K. algicida*, and co-cultures were incubated at 80°C for 10 min. The algicidal activity was monitored after inoculating 375 µL of *S. costatum* culture in 1.125 mL of regular or heat treated filtrate in 24 well plates.

### Conditioning of active filtrates

Replicates each containing 1.125 mL of active filtrate were inoculated with 375 µL of *S. costatum*, *C. didymus* or seawater in 24 well plates and incubated using the previously described culturing conditions. After 24 h each treatment was filtered through a 0.22 µm PES filter and the replicates within one treatment were combined. Aliquots of 1.125 mL of the combined filtrates were used to incubate 375 µL of exponentially growing *S. costatum* in 24 well plates. Other aliquots of the cell free filtrates were heat deactivated as described previously and inoculated with *S. costatum* in the same way to serve as controls.

### Protease inhibition experiment

Cell free bacterial filtrates were harvested as described above and the irreversible serine-protease inhibitor phenylmethanesulphonylfluoride (PMSF; Sigma, Munich, Germany) was tested for its ability to reduce algicidal activity against *S. costatum*. A working stock solution (1 M in isopropanol) was used to add a final concentration of 1 mM to active *K. algicida* filtrates and artificial seawater which was used as positive control. After incubation for 30 min in the dark at 15°C the filtrate was applied to *S. costatum* as described above and algal growth monitored as *in vivo* chlorophyll a fluorescence.

### Protease activity

The measurement of protease activity in bacterial filtrates was based on the conversion of BODIPY FL (E 6638) to a fluorescent product [30]. The dye was purchased from Invitrogen (Carlsbad CA, USA) and the assay was performed following the manufacturer’s instructions. Briefly, 10 µL of cell free filtrate of *K. algicida* cultures were diluted in 100 µL digestion buffer (Invitrogen) and 100 µL of the dye at a concentration of 10 µg mL^−1^ were added. After incubation at room temperature under exclusion of light for 1 h, the fluorescence was measured with a Mithras LB plate reader with an excitation filter of 470±5 nm and an emission filter of 510±20 nm. Linearity was ensured in independent calibrations.

### Calculation of protease release rate

The protease release rate (PRR) was calculated according to PRR  =  Δ(protease fluorescence)/(OD(Av) * Δ(t) where Δ(protease fluorescence) represents the difference between the measured fluorescence at two time points, OD(Av) represents the average of the OD at these time points and Δ(t) the time in hours between these time points. PRR values not significantly different from 0 are not displayed in the figures.

### Induction of protease release by conditioned bacterial filtrates

*K. algicida* was inoculated into 100 mL of a 10∶1 mixture of artificial seawater and ZoBell medium in three replicates. The growth and the protease release rate were regularly monitored until the first significant protease release was measured. Afterwards the cultures were sterile filtered, the cell free filtrate was pooled and proteases as well as other high molecular weight constituents were removed using Amicon Ultra centrifugal filter units. A volume of 10 mL of filtrate was added to *i*) 10 mL of freshly inoculated *K. algicida* in 1∶10 mixture medium and *ii*) to 10 mL *K. algicida* cultures inoculated 16 h before the addition of the conditioned filtrate. Protease activity in these inoculations was monitored as described above.

### Extraction of homoserine lactones

The attempt to extract acyl-homoserine lactones was performed with cell free supernatant of dense bacterial cultures. The supernatant was extracted with CH_2_Cl_2_ according to an established protocol [31] and samples were run on an Perkin Elmer Auto System XL gas chromatograph (GC) equipped with a SPB-5 column (40 m, 0.32 mm internal diameter and 0.25 µm film thikness. He 5.0 was used as a carrier gas with a constant pressure of 160 kPa. The GC was coupled with a Perkin Elmer TurboMass mass spectrometer (Waltham, MA, USA).

### Statistical analysis

The test for statistical significant differences at different time points over the course of an experiment was conducted using a two way repeated measures analysis of variance (RM-ANOVA) with Sigmaplot 11. Post hoc test of significance was performed using the Tukey method implemented in Sigmaplot 11. A student t-test was performed to exclude PRR values that were not significantly different from 0. Significance level was generally set for all analysis P<0.05.

## Results

### Effect of K. algicida and cell free filtrates on different diatom species

**Figure 1:**
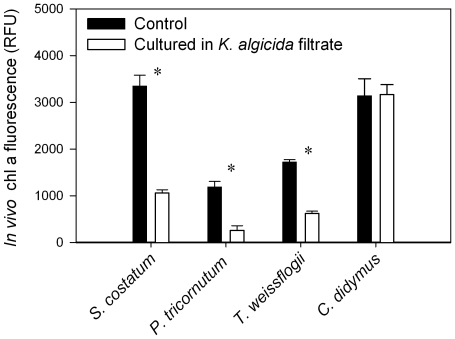
Screening for the susceptibility of four different diatom species to *K. algicida*filtrate. Mean values of *in vivo* fluorescence + SD (n = 4) displayed are measured after 39 h. Asterisks indicate significant differences between the respective control and treatment.

In an initial experiment we prepared a co-culture of *S. costatum* and *K. algicida* and monitored the cell growth of *S. costatum*. We observed a significant reduction of the diatoms cell density after 7 h (P = 0.04). After 25 h the cell density of the co-cultured diatoms was only 12.1% of the corresponding control (data not shown). All further experiments were performed with cultures of this active *K. algicida*. We tested the effect of a cell free filtrate of *K. algicida* on the diatoms *S. costatum*, *C. didymus*, *P. tricornutum* and *T. weissflogii* over the period of 64 h. Fig. 1 shows the *in vivo* chlorophyll a readings 39 h after inoculation. The cell growth of *S. costatum*, *P. tricornutum* and *T. weissflogii* were significantly inhibited (P<0.001 for all species). A two way repeated measures ANOVA revealed significant differences between treatment and the corresponding controls for all data points recorded 24 h after inoculation or later (P<0.001 for all species, data not shown). At the end of the experiment (t = 64 h) the *in vivo* chlorophyll a fluorescence in the treatments were only 8.7%, 8.7% and 19.4% of the respective control for *S. costatum*, *P. tricornutum*, and *T. weissflogii* respectively (data not shown). In contrast, *C. didymus* growth was not affected by *K. algicida* filtrate (P≥0.553 at all sampling points; RM-ANOVA). At the end of the experiment the *in vivo* chlorophyll a signal in the treatment was 99.7% of the corresponding control.

### No regulation of algicidal activity in the presence of the host

**Figure 2:**
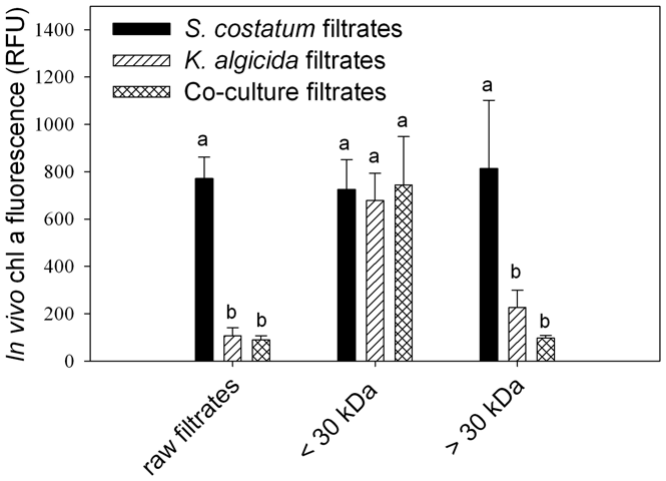
Effects of size fractionated *S. costatum*, *K. algicida* and co-culture cell free filtrates on the susceptible species *S. costatum* determined by *in vivo* fluorescence. Values displayed are measured after 47 h and are mean values + SD (n = 5 for all but *S. costatum* where n = 4). Different letters indicate statistically significant differences.

The filtrate of active *K. algicida* cultures as well as the filtrate of *K. algicida*/*S. costatum* co-cultures both caused a significant decrease of cell growth of *S. costatum* in comparison to *S. costatum* filtrate as control (P<0.001 for both) (Fig. 2). No up-regulation of algicidal activity was observed in the presence of *S. costatum,* since the effect of the filtrate of *K. algicida* and of *K. algicida*/*S. costatum* co-cultures did not differ significantly (P = 0.821).

### Size fractionation of the bioactive filtrate

The filtrates of *K. algicida* and co-cultures of *K. algicida* and *S. costatum* containing only compounds with a molecular weight below 30 kDa had no inhibitory effect compared to the corresponding control using a <30 kDa fraction of medium from a *S. costatum* culture (P = 1 for both) (Fig. 2). None of these treatments were significantly different from a treatment with an unfractionated filtrate of a *S. costatum* culture (P≥0.899 in all cases). In contrast, treatments with the high molecular weight fraction >30 kDa of the *K. algicida* and co-culture filtrates resulted in a significant inhibition of algal growth in comparison to the control (P<0.001 for both). The inhibition caused by the high molecular weight filtrate of the *K. algicida* culture was not significantly different from the inhibition by the high molecular weight filtrate of the co-culture (P = 0.832). The effects of high molecular weight filtrates of both *K. algicida* cultures and co-cultures were not significantly different as compared to the effect of the corresponding unfractionated filtrates (P> 0.321 for all comparisons) (Fig. 2).

### Heat deactivated filtrates

The filtrates of *K. algicida* and co-cultures of *K. algicida* and *S. costatum* significantly inhibited the growth of *S. costatum* while aliquots of the same filtrates that were heated at 80°C for 10 min prior to the assay had no negative effect for any monitored time point (data not shown) (P<0.001 for a comparison of the effect of filtrates versus heat treated filtrates at t = 38 h onwards). Filtrates of *K. algicida* and of the co-cultures again showed no significant difference in their activity over the complete time course of this experiment (P>0.982).

### Protease as the inhibiting enzyme

**Figure 3:**
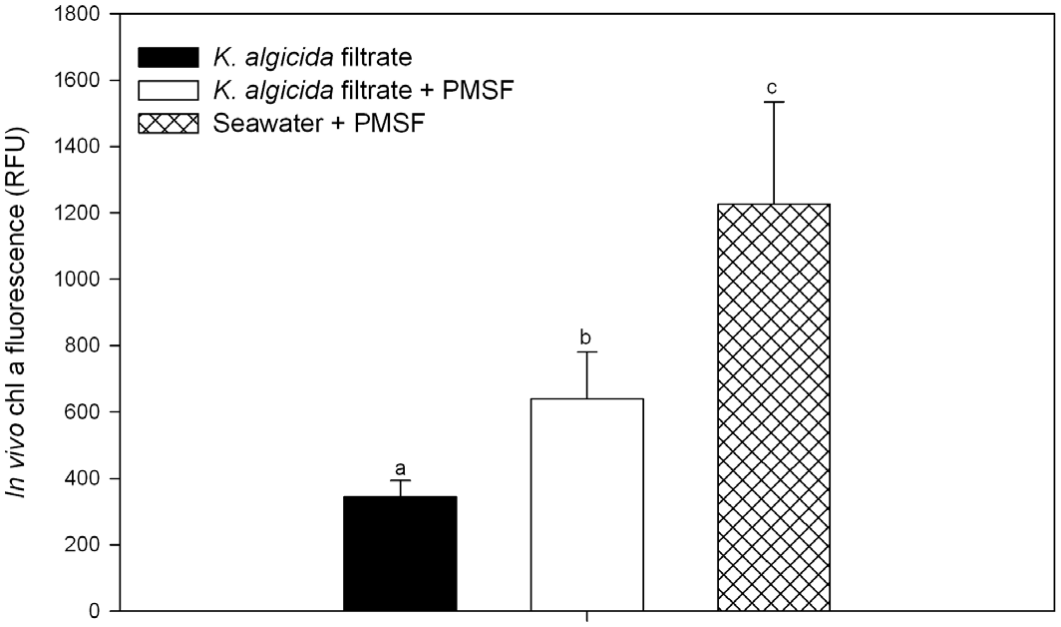
Effect of the protease inhibitor PMSF on the inhibiting effect of *K. algicida* filtrates. Values displayed are measured after 52 h and are mean values + SD (n = 5).

Aiming to identify the inhibiting activity of *K. algicida* we performed experiments adding commercially available protease from *Streptomyces griseus* to *S. costatum* and *C. didymus* in a concentration range of 1.7 U mg^−1^ to 0.2 U mg^−1^. While *C. didymus* was not affected by any of these protease additions *S. costatum* was inhibited in growth by the external proteases (data not shown). Further evidence for the involvement of proteases in the interaction was gained by protease inhibition experiments. The addition of the serine-protease inhibitor phenylmethanesulphonylfluoride (PMSF) significantly decreased the inhibition of algal growth by *K. algicida* medium in comparison to controls without PMSF (P<0.038). However, the protease inhibitor did not re-establish the complete algal growth and resulted in significant less *in vivo* chlorophyll a fluorescence compared to a seawater control (Fig. 3) (P<0.001).

### Test for detoxification of the K. algicida activity by *C. didymus*

**Figure 4:**
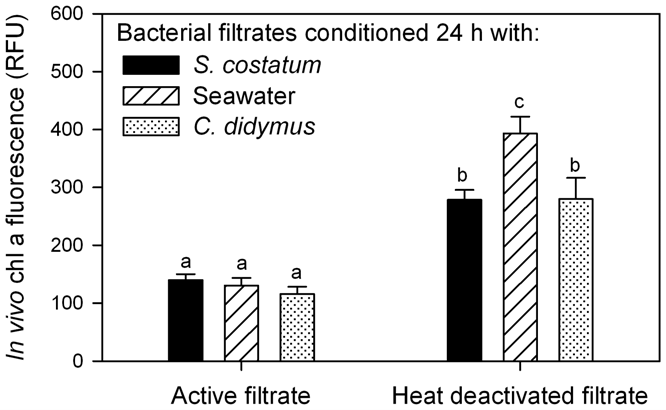
Left: Growth of *S. costatum* indicated as *in vivo* fluorescence in *K. algicida* filtrate conditioned for 24 h with *S. costatum*, *C. didymus* or seawater. Right: Incubations with the same filtrates that were heat inactivated before the start of the experiment. Displayed are mean values + SD (n = 6) taken 70****h after the start of the experiment. A statistically significant difference is indicates by different letters above the bar.

An active filtrate of *K. algicida* was incubated for 24 h with a *C. didymus* culture to test whether *C. didymus* could deactivate algicidal activity. As controls aliquots of the same *K. algicida* filtrate were used without further treatment or after incubation with a *S. costatum* culture. After a second filtration to remove the diatoms, the respective filtrates were used in incubations with *S. costatum*. Neither incubation of the active filtrate with *C. didymus* nor with *S. costatum* resulted in decreased activity as compared to the control (Fig. 4) (P≥0.956 and P≥0.585, respectively over the entire time of the experiment). To test if this effect was due to a general loss of activity all three filtrates were heat inactivated, resulting in significantly reduced activity in all cases (P<0.005 for all comparisons 54 h and onwards).

### Protease release by *K. algicida*

**Figure 5:**
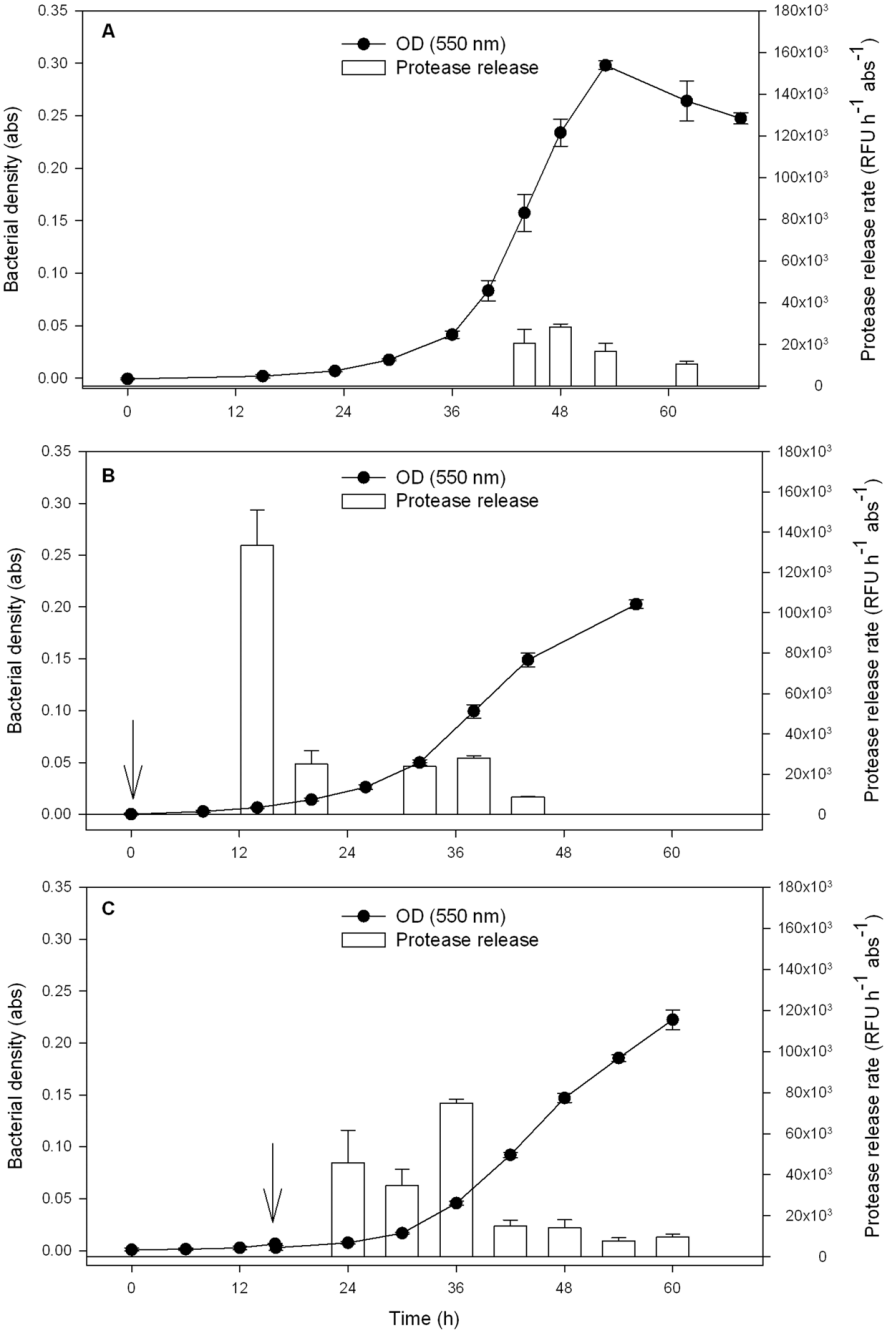
Protease release by *K. algicida* during the growth of a culture. A) Protease release pattern of *K. algicida* under standard growth conditions. B) Protease release pattern of *K. algicida* with conditioned cell free filtrate added directly with the inoculation of the cultures. C) Protease release pattern of *K. algicida* with conditioned cell free filtrate added 16 h after the inoculation of the cultures. The line indicates bacterial growth measured as OD and the bars give the bacterial protease release rate. The arrows indicate the time of the addition of *K. algicida* conditioned cell free filtrate. Displayed are mean values + SD (n = 3).

Exponential bacterial growth started after a lag period of 29 h. During this period there was no detectable protease activity in the *K. algicida* medium (Fig. 5A). After 29 h the culture started to grow exponentially and reached a growth rate of µ = 0.142±0.004 h^−1^. Exponential growth lasted from t = 29 until t = 48 h. In the beginning of the exponential growth phase there was no protease release. A significant release of proteases started after 44 h, in the late exponential phase. This release proceeded for 18 h and stopped after 62 h. In later stationary growth we could not observe any protease release. To exclude an underestimation of the protease release due to potential instability of the enzyme, we verified the protease stability in seawater over a period of 9 h. After this time period no detectable decrease of the protease activity could be observed (P = 0.866 in student t-test, data not shown).

### Induction of protease release

In order to test if chemical communication regulates bacterial activity as known from quorum sensing we examined the effect of cell free bacterial filtrate on the excretion of protease from freshly inoculated *K. algicida* cultures and cultures that were incubated for 16 h (Fig. 5 B & C). The addition of *K. algicida* conditioned cell free filtrate to freshly inoculated bacteria cultures accelerated the protease release. These cultures exhibited already a significant protease release rate after 14 hours of cultivation (Fig. 5B) which was approximately 5 times higher than the release rate observed under standard growth conditions (Fig. 5A). Under standard cultivation conditions an optical density of >0.1 was needed before protease release occurred. In contrast cultures where conditioned cell free medium was applied started to excrete significant amounts of protease already at an optical density <0.01 (Fig. 5B). This protease release was stopped after 26 h and started again after 32 h when an optical density of >0.05 was reached.

If the same cell free filtrate was added to *K. algicida* cultures 16 h after inoculation we observed a significant release of bacterial proteases already after 8 h confirming an induction of enzyme release by a bacterial cell free filtrate (Fig. 5C).

## Discussion

The marine bacterium *K. algicida* has a strong algicidal effect on the diatom *S. costatum*. In a direct contact situation a significant inhibition of diatom proliferation can be observed after 7 h if a dense bacteria culture is employed for incubation. This is consistent with previous findings of *S. costatum* cells that were killed quantitatively after 3 days in a co-culture with *K. algicida* [27]. The negative effect of the bacterium is not exclusively transmitted through contact with the diatom but can be also mediated via diffusible compounds. This is clearly demonstrated by the fact that activity of the *K. algicida* medium remains after removal of the cells by sterile filtration. Inhibition of growth relative to a control is observable within the first 24 hours of incubation, indicating rapid action of the algicidal compounds. Compared to reports from other systems, where a>24 h delay of effects on the algal cells was observed after algae were treated with algicidal bacteria, both the direct interaction as well as the action of the filtrate reported here are quick [8], [32]. Diffusible substances mediating algicidal activity have been previously observed from bacteria and can include both, small molecular weight metabolites as well as proteins [33], [34]. The use of dissolved substances to inhibit the growth of algae is common in bacteria belonging to the phylum of γ-proteobacteria which includes the genera *Alteromonas* [8], *Pseudoalteromonas* [22], [35] and *Vibrio* [36]. However, *K. algicida* belongs to the *Cytophaga-Flavobacterium-Bacteroides* phylum (CFB). Genera within this group usually require direct cell contact to kill their prey [16], [35], although there are exceptions reported [18]. *K. algicida* is thus a rare example of a CFB bacterium that does not require cell contact with its prey to inhibit the algal growth, but releases diffusible active enzymes.

The release of active substances by *K. algicida* allowed us to further explore the nature of the active principles. A first survey revealed that *K. algicida* filtrate is also active against other diatom species (Fig. 1). The activity against the pennate diatom *P. tricornutum*, as well as that against the centric diatom *T. weissflogii* was comparable to that observed against *S. costatum*. In contrast, another centric diatom, *C. didymus* was not susceptible against the diffusible factors released by *K. algicida.* This missing susceptibility is apparently not due to an active detoxification by *C. didymus* since medium from a *C. didymus*/*K. algicida* co-culture is still active against *S. costatum* (Fig. 4). The physiological properties which mediate *C. didymus* resistance cannot, however, be concluded from our experiments. Selectivity of algicidal activity is important to understand ecological interactions within the planktonic community. Additionally, proposals to apply bacteria in order to control red tides should seriously consider the selectivity of algicidal activity [35], [37]. Different levels of specificity have been observed from algicidal bacteria. Selective activity against one algal species and universal activity against all tested species in a given taxon have been reported as well as all intermediate forms of specificity like they are shown here [16]. From an ecological perspective it is obvious that resistance mechanisms of algae have the potential to provide selective advantages. When other diatom species that are potential competitors for resources are inhibited, the unsusceptible alga can proliferate. Thereby the bacteria can directly influence plankton species successions.

Basic characterization of the released algicide showed that it bears all hallmarks of an enzyme. It has a molecular weight >30 kDa (Fig. 2) and the activity can be inactivated by heat treatment. A survey of the literature suggests that dissolved proteases are prime candidates for algicidal enzymes. Lee et al. were the first to demonstrate the activity of proteases in the interaction of the bacterium *Pseudoalteromonas* sp. and the diatom *Skeletonema costatum* [20]. After indirect evidence from bioassays they isolated a 50-kDa serine protease with algicidal activity. Several subsequent studies supported the role of enzymes from algicidal bacteria in the lysis of algae [17]. Using fluorescence based assays we were able to show that active medium from *K. algicida* and from *K. algicida/S. costatum* co-cultures exhibited substantial protease activity. Indeed, *S. costatum* was susceptible to protease treatment. If the protease from the bacterium *Streptomyces griseus* was applied the diatom growth was inhibited compared to a control. In agreement, application of the protease inhibitor PMSF to active *K. algicida* medium resulted in a significantly higher growth of *S. costatum* compared to uninhibited controls. The growth of *S. costatum* was, however, not fully restored after the application of the protease inhibitor. Similarly, PMSF did not fully neutralize the motility reduction of the dinoflagellate *Lingulodinium polyedrum* caused by bacterial proteases [38]. The inhibitor experiment demonstrates, however, the involvement of a protease in the interaction but it might well be that additional activities can be responsible for the observed interactions. Alternatively, the algicidal protease might not be very sensitive to the inhibitor PMSF and the applied concentration might not be sufficient for a quantitative inhibition.

It has been argued that the release of a freely diffusible algicide is unlikely to be energetically efficient for killing algal cells suspended in seawater [16]. Since ratio of the volume of bacterial cells to the volume of seawater they inhabit is ca. 10^−7^ in an average dilute situation in the plankton [39] an uncontrolled release of any active principle would most likely not result in concentrations sufficient for algicidal activity or result in high costs. However, a release of active metabolites could provide a selective advantage if it is under the control of a metabolic switch that is triggered only under environmental conditions where the production of algicides is beneficial. We tested the hypothesis that algicidal activity is only induced in the presence of susceptible algae or in the presence of signals of these algae. No evidence was found for such an induced mechanism since algicidal activity did not increase in the presence of diatoms (Fig. 2).

Another possibility to increase the success of released active compounds would be a metabolic switch dependent on the density of a bacterial population. Based on the findings that the algicidal activity observed in our study was caused by a protease, we monitored protease activity as a function of *K. algicida* culture density. We indeed observed a synchronized release of a protease, which could be explained by quorum sensing like mechanism in *K. algicida* (Fig. 5) [40]. We found support for such phenomenon using experiments with conditioned cell free supernatant of *K. algicida.* After adding such filtrates to freshly inoculated *K. algicida* cultures the protease release was remarkably accelerated (Fig. 5). These results fit to known quorum sensing dependent secretion mechanisms of other bacteria species such as the human pathogen *Pseudomonas aeruginosa* where the excretion of exoenzymes that determine virulence is controlled by bacterial density [41], [42]. Quorum sensing in gram negative bacteria is often mediated by acyl homoserine lactones (AHL) as it can be for example observed in the *P. aeruginosa* pathogenicity [41], [42]. We were, however, not able to detect any AHL in dichloromethane extracts of protease releasing cultures using sensitive GC/MS methods. Several other QS molecules that have been previously described for Gram-negative bacteria can be considered as alternative candidates and further tests will have to be performed in the search for the regulative principle in plankton assemblages [43], [44]. Gram-negative bacteria found in all kinds of habitats often rely on quorum sensing signals to trigger metabolic events. In planktonic bacteria the alternative induction pathway (AI-2) for quorum sensing type regulation has been detected although it could not be directly linked to algicidal activity [18]. Evidence also exists for the QS-regulation of the production of the algicidal pigment PG-L-1 in a marine *γ-proteobacterium* [45]. While these studies give rather indirect evidence we can show here clearly that release rates of active principles are regulated. Comparable regulative mechanisms have also been suggested in a study of algicidal *Pseudoalteromonas* sp.. Mitsutani et al. [17] could show in gel electrophoretic experiments that the production of several enzymes was only observed during stationary phase and that bacteria only exhibited algicidal activity during this phase.

In the plankton such a density dependent release of proteases might provide an advantage if a sufficient bacterial density is required for efficient lysis of algae. Diffusible substances aiding algal lysis might provide a benefit for locally dense bacterial assemblages. Bacteria could jointly overcome defense systems of the alga in cases when active principles from single bacteria would not be effective. Lysis of algal cells could increase available nutrient concentrations in the vicinity of the bacterial assemblage and such a control could be an efficient means for a concerted mobilization of resources.

Our results on the specificity of the algicidal activity as well as on the density dependent regulation of the release of an active protease by an algicidal bacterium support the view that a multitude of chemical signals can regulate plankton interactions on all levels.
